# Cardiac Biomarkers in Patients with Cancer: Considerations, Clinical Implications, and Future Avenues

**DOI:** 10.1007/s11912-020-00930-x

**Published:** 2020-06-09

**Authors:** Valentina Bracun, Joseph Pierre Aboumsallem, Peter van der Meer, Rudolf A. de Boer

**Affiliations:** Department of Cardiology, University Medical Center Groningen, University of Groningen, AB31, PO Box 30.001, 9700 RB Groningen, the Netherlands

**Keywords:** Biomarkers, Chemotherapy, Cardiotoxicity, Heart failure, Cardio-oncology

## Abstract

**Purpose of the Review:**

As the number of cancer survivors increases due to early screening and modern (antineoplastic) treatments, cancer treatment associated cardiotoxicity (CTAC) is becoming an increasing health burden that affects survival and quality of life among cancer survivors. Thus, clinicians need to identify adverse events early, in an effort to take suitable measures before the occurrence of permanent or irreversible cardiac dysfunction.

**Recent Findings:**

Cardiac troponin (cTn) and B-type natriuretic peptide (BNP) have been proven to detect subclinical cardiotoxicity during antineoplastic treatment. As such, these cardio-specific biomarkers could predict which patients are at risk of developing CTAC even before the start of therapy. Nevertheless, there are inconsistent data from published studies, and the recommendations regarding the use of these biomarkers and their validity are mostly based on expert consensus opinion.

**Summary:**

In this review, we summarize available literature that evaluates biomarkers of CTAC, and we encourage strategies that integrate circulating biomarkers and cardiac imaging in identifying cancer patients that are at high risk.

## Introduction

Now that efforts to prevent cardiovascular disease (CVD) have improved outcomes, cancer is rising as the major cause of mortality and morbidity in high-income countries [1]. According to the World Health Organization, Europe, which represents 9% of the global population, accounts for 23.4% of the global cancer cases and 20.3% of global cancer deaths. There are approximately 8.7 million cancer survivors in Europe, [2] and more than 3 million new cases are predicted to occur every year [3]. The Americans come in the second place with 21% and 14.4% of the global incidence and mortality, respectively [4]. According to the American Cancer Society, around 17 million Americans have a history of cancer and the number of cancer survivors is expected to increase to more than 22.1 million in 2030 [5]. Furthermore, cancer and CVD share multiple risk factors and in fact very often coincide [6, 7].

In the last years, cancer management and treatments have improved. However, the use of both traditional and novel antineoplastic agents is limited by their toxic effects, often leading to CTAC. Previously, the risk of CTAC was not recognized because the life span of patients with a neoplasm was too short to make CVD a major concern. Today, the progress in early diagnosis and the advances in cancer therapies have resulted in a prolonged life expectancy and improved survivorship. Consequently, the medical community recognizes the CTAC in response to antineoplastic agents as a clinically relevant issue, which has been highlighted in several recent articles [8–11, 12•].

Since functional deteriorations only become clinically manifest in later stages, when cardiotoxicity becomes irreversible, physicians should focus on early detection of CVD when treatment or prevention may still be an option [13]. Regarding the diagnosis of CVD, many biomarkers have demonstrated the ability to predict cardiac dysfunction before the occurrence of clinical signs or symptoms. However, the progress in the CVD biomarker field has been slow considering the fact that a good biomarker must satisfy strict criteria, including a solid and easy use of assays, good sensitivity, specificity, and knowledge on the confounders and the context of the assay. Although for several biomarker assays, these requirements are satisfied, in the setting of CTAC, this is often less straightforward. The lack of unified clinical guidelines leaves clinicians dependent on their own judgment and generalized expert opinions.

Herein, we discuss the current and future role of circulating biomarkers in the assessment of the CTAC. This review encourages to establish recommendations and guidelines on the use of circulating biomarkers and urges further research on their contribution to the pathophysiology and mechanisms mediating the CTAC. Furthermore, we would like to stress out that the old definition of cardiotoxicity does not fit the adverse effects of newer antineoplastic treatments. The revision and new systematization of CTAC are needed to meet the requirements of clinicians working in cardio-oncology practices and to better correspond with research endpoints.

## Cancer, an Independent Risk Factor for CVD

Although most focus is on CTAC, there are also direct effects of cancer on the CV system. Eighty percent of all cancer patients show significant loss in muscle strength and weight, including muscle cells of the heart [14]. More than 50 years ago, Burch et al. noticed that patients who died of cancer had smaller hearts, reduced total mass, and smaller left ventricular (LV) wall thickness [15]. Cancer cells alter metabolic pathways by inducing the catabolic state in muscle cells, which leads to cardiac cachexia [14]. More recently, Pavo et al. reported that circulating cardiac biomarkers NT-proBNP and hsTnT increased with advancing tumor stage even before starting antineoplastic treatments [16]. Increased biomarker levels were strongly related to all-cause mortality. Furthermore, it has been reported that atrial natriuretic peptide (ANP), aside from its known potent diuretic, natriuretic, and vasodilatation effects, also has several immune functions [17]. It stimulates the immune system in defense against microbes, counteracts reactive oxygen species effects, and inhibits tumor growth by inducing apoptosis and necrosis. Furthermore, it has been reported that ANP and several other cardiac hormones such as long-acting NP can inhibit several tumor cell lines. Meijers et al. went a step further and showed that failing heart serves as a secreting organ that stimulates tumor growth [18•]. Recently, Shi et al. also reported a correlation between several tumor biomarkers and heart failure, showing that tumor biomarkers have an independent prognostic value in heart failure patients [19]. This link also suggests a strong overlap and possible direct pathophysiological mechanism connecting CVD and cancer [6, 7, 20]. Therefore, the relation between cancer and heart failure appears much stronger than initially thought.

## Biomarkers for the Diagnosis of CTAC

The early diagnosis of patients who are at increased risk and with asymptomatic CTAC is required to establish adequate preventive and therapeutic strategies. This could result not only in prolonged survival but also, in improved quality of life. However, cardiac follow-up based on clinical signs and symptoms of heart failure will likely result in missing a substantial proportion of patients in the early stage of cardiac dysfunction [21]. Furthermore, with the increasing trend of new antineoplastic treatments and new combination therapies, where short- and long-term complications remain peculiar, it is extremely challenging for a clinician to promptly and adequately diagnose CTAC.

**Fig. 1:**
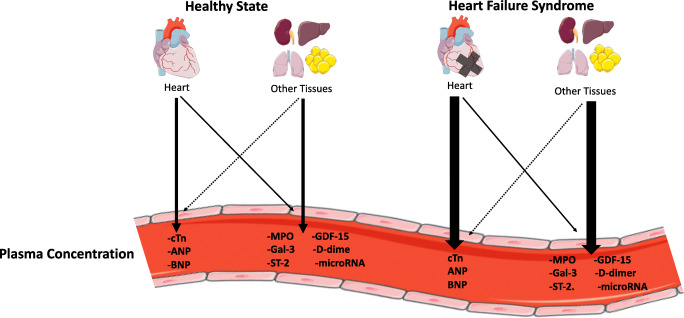
Theoretical paradigm of the heart and other tissues contribution to plasma biomarkers levels. The larger arrow signifies a stronger relative contribution [23]. Illustration elements are from Smart Servier Medical Art

In the following section, we discuss the widely studied biomarkers: cTn and natriuretic peptides, such as BNP or its stable precursor NT-proBNP. These two families are cardio-specific, which means that these proteins have been produced or released exclusively by the heart. The actual plasma levels may nevertheless be confounded by several well-known factors such as age, sex, impaired renal function, obesity, and other co-morbid conditions, such as cardiac ischemia, pulmonary embolism, and arrhythmia. Clearly, there are numerous novel biomarkers, but none of these have made into daily care [22]. This has to do with the fact that these markers are mostly not cardio-specific. We have recently shown that novel biomarkers, such as Galectin-3 (Gal-3) and growth differentiation factor (GDF)-15 are to be considered as extra-cardiac biomarkers [23]. In other words, the majority of the production is extra-cardiac, and increased plasma levels will confer information of all organs producing such factors, including tumors (Fig. 1).

### Cardiac Troponins, Predictors of CTAC

**Fig. 2:**
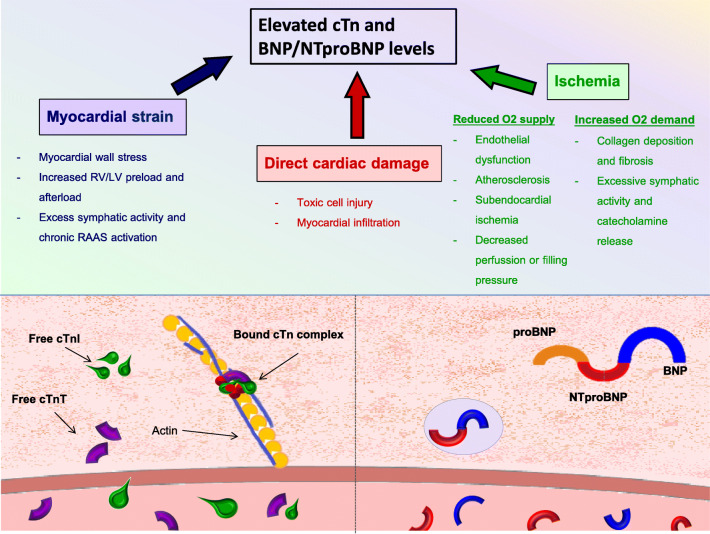
The pathophysiological mechanisms of cardiotoxicity. Diverse pathophysiological mechanisms are known to cause CTAC. They are also known to cause elevated cardiac troponin (cTn) and natriuretic peptides (BNP/NTproBNP levels)

In clinical practice, cTn is routinely measured in acute myocardial infarction (MI) for diagnostic and prognostic purposes [24]. Elevated cTn levels (according to the acute coronary syndrome threshold) are commonly found in conditions that either cause direct myocardial damage, excessive myocardial strain, or myocardial ischemia based on increased O_2_ demand or reduced O_2_ supply [25]. Also, cTn levels have been shown to have predictive value for most CVDs, including hypertension and (stable) coronary artery disease (CAD) in seemingly healthy individuals [26]. Several pathophysiological mechanisms in CTAC, such as oxidative stress, cardiac inflammation, fibrosis, and plaque rupture or even thrombosis, lead to direct myocardial damage, and direct micro- and macro-vascular impairments, which results in myocardial ischemia, and activation of the immune system (Fig. 2). Conventional and high sensitivity (hs)-cTn assays have shown prognostic values in chronically ill individuals with CVD, diabetes mellitus, chronic kidney disease, anemia, and stroke [25, 27], where elevated cTn levels have also been frequently reported [28, 29]. Of note, such conditions are frequently observed in the cancer population, which makes it even more difficult to distinguish chemotherapy induced from classical cTn elevations [29]. cTn has been extensively studied in patients with cancer (Table 1). Ky et al. examined the possible relationship between the release of several circulating cardiac biomarkers and cardiotoxicity in patients with breast cancer receiving anthracyclines and trastuzumab [34]. The selected biomarkers were known to be involved in CTAC pathophysiological mechanisms, such as myocardial injury (cTnI), inflammation (CRP), neurohormonal activation (NT-proBNP), oxidative stress (Myeloperoxidase (MPO), and fibrosis (Gal-3). The investigators found that elevation in absolute cTnI levels was associated with an increased risk for CTAC. This study showed that elevations in 2 or more biomarkers, in this case, cTnI and MPO, were associated with a higher risk of CTAC. Besides cTn and NT-proBNP, all the biomarkers used in the study were not cardio-specific, but their combination showed improved sensitivity. Blaes et al. focused their research on hs-cTn elevations in patients receiving anthracyclines [31]. They demonstrated a significant correlation between elevated baseline hs-cTn levels and the risk of developing CTAC. The authors also reported an increased risk of elevated cTn levels for *asymptomatic left ventricular* systolic dysfunction (LVSD) that did not meet the (strict) CTAC criteria. Their conclusion was that elevated hs-cTn levels are often observed after anthracycline treatment, not only in patients with increased CV risk but also in low-risk individuals. Their conclusion is one of great clinical relevance, showing that myocardial damage and therefore cTn elevations following antineoplastic treatment can be more frequently observed than initially thought. In line with this, Zardavas et al. reported an increased risk of trastuzumab-related cardiotoxicity (TRIC) in patients with increased cTn levels after anthracycline treatment [32]. This could be explained by the increased myocardial susceptibility to damage when the combination of anthracyclines and trastuzumab is being used [45, 46]. On another hand, Kitayama et al. reported elevated absolute hs-cTn levels throughout the treatment in patients developing CTAC, while baseline hs-cTn levels did not seem to have a significant predictive value [35]. Even more compelling is the author’s advocacy of a different approach to circulating biomarkers when it comes to predicting CTAC. They suggest that the integration of baseline values with hs-cTn increments could be more reliable in predicting CTAC compared with absolute hs-cTn values. The predictive value of hs-cTn is consistent among other neoplasm patients as well. Zhang et al. followed patients with B cell lymphoma receiving anthracyclines [33]. They have also reported a significant hs-cTn rise following anthracycline treatment. Those values were more prominent in patients with CTAC. Interestingly, elevated hs-cTn levels were also predictive of left ventricular diastolic dysfunction (LVDD). The authors advocated both the use of echo parameters and circulating cardiac biomarkers in predicting CTAC, seeing their combination significantly improves sensitivity. Mahjob et al. have also emphasized the usefulness of combining circulating cardiac biomarkers with echo parameters in detecting CTAC [30]. The authors have made several important conclusions and suggested a clear correlation between elevated baseline cTn level and increased risk of CTAC, making the case for adequate CV risk prevention and therapy even stronger. Additionally, significant cTn elevations after the treatment with anthracyclines were also predictive of the increased CTAC risk. Demissei et al. focused their research on patients with breast cancer [43]. What makes this study so compelling is the recognition of different pathophysiological mechanisms behind anthracyclines, trastuzumab, and their combination. The authors reported that cTn elevations i.e., cardiac injury, can frequently be expected after anthracycline treatment. However, only persistently elevated cTn levels at the time of completion of anthracycline treatment, and not the baseline levels showed to be predictive of CTAC. Similarly, elevated baseline MPO levels in patients receiving anthracyclines and trastuzumab could be predictive of CTAC (HR per doubling 1.28; 95% CI, 1.04–1.57, *P* = 0.019).

**Table 1:** The timing of the circulating cardiac biomarker rise in response to cancer treatment. Summary of human trials dealing with cardiotoxicity and circulating cardiac biomarkers in the last 5 years

**Cancer type**	**Mean ANT exposure**	**Reported CTx*N*(%)**	**Biomarker**	**Reported time of significant elevation (days)**
Breast, leukemia, lymphoma [30]	240–360 mg/m^2^ DOX	5 (9.6)	hs-cTnI	21
Breast, NHL [31]	240–402 mg/m^2^ DOX	0	hs-cTnT	91–154
Breast [32]	Not stated	3 (0.6) + 31 (7)	hs-cTnT, hs-cTnI	90
Lymphoma [33]	496.2 ± 89.4 mg/m^2^–707.9 ± 83.0 mg/m^2^ DOX	5 (6)	hs-cTnT	42–84
Breast [34]	240 mg/m^2^ DOX	23 (24)	cTnI	90
Breast [35]	300–400 mg/m^2^ EPI	4 (10)	hs-cTnT	90
Breast [36]	240 mg/m^2^ DOX	Not stated	hs-cTnT	2–22
Breast, NHL, leukemia [37•]	240 mg/m^2^ DOX, 360 mg/m^2^ EPI	3 (1)	cTn	25
Breast [38•]	240–400 mg/m^2^ EPI	1	hs-cTnI, hs-cTnT	77–87
Breast [39•]	240 mg/m^2^ DOX	27 (14)	hs-cTnI	84
Lymphoma, sacoma, breast [40]	308 ± 111 mg/m^2^ (not specified)	11 (10)	NTproBNP	108
Breast [41]	0	4 (9)	NTproBNP	30–180
Breast [42]	Not stated	45 (33)	NTproBNP	Not stated
Breast [43]	240 mg/m^2^	57 (22%)	hs-cTnT, NT-proBNP	0–180 (for both)
Lymphoma, myeloma [44]	0	12%	hs-cTnT	16

*ANT* anthracyclines, *DOX* doxorubicine, *EPI* epirubicine, *CTx* cardiotoxicity, *NHL* non-Hodgkin lymphoma, *hs-cTnI* high sensitive cardiac troponin I, *hs-cTnT* high sensitive cardiac troponin T

Nevertheless, frequent CV risk assessment and cardiac follow-up may be favorable for the early and prompt diagnosis of CTAC for other cancer treatments, e.g., in patients receiving immune checkpoint inhibitors (ICI). Their pathophysiological mechanisms cause a wide spectrum of immune-related adverse effects including cardiac ones, (IRCAE), such as myocarditis, pericarditis, and vasculitis. IRCAE are mostly known as early adverse effects that occur in the first 90 days from the start of the treatment. Cases of fatal myocarditis are being more frequently described and it has also been suggested that its incidence is underestimated [47–49]. Mahmood et al. reported that nearly all patients (94%) with ICI-related myocarditis had cTn elevations. In the same time, only 49% of the patients had reduced LVEF [47]. Seeing the high mortality rates of nearly 50%, the baseline value and continuous cTn monitoring throughout the cancer treatment become even more crucial [50]. Furthermore, concerns have been raised about late IRCAE occurring 90 days after the start of ICI treatment [51]. Patients diagnosed with late IRCAE mostly show symptoms and signs of heart failure and not myocarditis. Since the different pathophysiological mechanisms determine early and late IRCAE, a different practical approach involving cardiac biomarkers (circulating and imaging) must be considered. Further research is needed to conclude the usefulness of cTn and possibly NT-proBNP in these patients. Close cardiac monitoring during, but also not any less significant, years following the cancer treatment should be considered after ICI treatment. Another, even more recent immune therapy treatment, chimeric antigen receptor T cells (CAR-T) also carry significant IRCAE risks. Alvi et al. reported that up to 59% of the patients experienced cardiac damage during the CAR-T treatment, of whom 4% had cardiac-related death [44, 52]. In their study, the investigators report that 94% of the CV events occurred in the patients with elevated cTn (again, according to ACS threshold). Interestingly, early treatment with tocilizumab resulted in significant reduction of CV events. The CV event risk increased by 1.7-fold for every 12-h treatment delay. The data validate once again the importance of IRCAE’s early diagnosis, and therefore the importance of frequent and consistent measurements of circulating cardiac biomarker.

### cTn in Preventive and Guided Therapies

Several other studies focused on the use of preventive and cTn-guided therapies. Gulati et al. focused their research on the possible beneficial effects of the angiotensin receptor blocker (ARB) candesartan cilexetil and the β-blocker metoprolol succinate on CTAC prevention in patients with breast cancer receiving anthracycline-based chemotherapy [38•]. The authors reported that candesartan significantly reduced the LVEF decline compared with the placebo. However, it did not affect cTn levels. Furthermore, metoprolol had no beneficial effect on the prevention of LVEF decline. A recent study from Cardinale et al. showed that despite preventive treatment with ACE inhibitors (enalapril), patients with higher anthracycline doses had elevated cTn levels throughout the treatment [37•]. Therefore, the authors supported the conclusion made by Gulati et al., which states that ARB/ACE inhibitors are less likely to prevent direct myocardial damage caused by anthracyclines (as they did not affect cTn levels), but they possibly have a beneficial effect on cardiac remodeling occurring as a result of CTAC, as they do reduce the overall CTAC incidence. Avila et al. explored the possible preventive role of another β-blocker, carvedilol in patients receiving anthracyclines [39•]. They randomized patients with breast cancer to receive carvedilol or placebo until chemotherapy completion. Their results showed that carvedilol did not affect the cardiotoxicity incidence but it reduced the surrogate outcomes, cTn levels, and LV end-diastolic diameter. At this moment, the lack of prospective data and the substantial amount of contradictory results reported in clinical trials make it impossible to draw robust conclusions on the beneficial effects of β-blockers and ARB/ACE-I in patients receiving chemotherapy. However, it is reasonable to support their use as these agents have demonstrated cardio-protective properties. Also, they are generally safe and already in use in several cardio-oncology clinics [39, 53, 54].

### cTns Assays and Considerations

One of the explanations for the broad diversity in available data may be explained by the fact that changes in conventional cTn assays, used in the first studies, were not sensitive enough to detect early, subclinical myocardial injury, as reported by Blaes et al. [31]. Second-generation cTns assays that measure high sensitivity (hs)-cTns, and are far more sensitive. With improved sensitivity, the possibility of optimizing the risk stratification and therefore cardiac safety, by measuring 5–10 times lower troponin concentrations have increased [55]. Nevertheless, several older studies reported an important role of elevated conventional cTn levels in detecting early myocardial damage due to chemotherapy [56–58].

Additional explanations could be the lack of gender and age-specific cutoff points. Recently, Rocco et al. reported that standard troponin values in younger patients and women were much lower compared with older patients and men [59]. This may be especially significant in breast cancer patients as it affects women of all ages (including the young) where high-dose protocols and combination chemotherapy treatments are used [38, 45, 60]. Moreover, it has also been postulated that sex hormones, specifically estrogen, could influence the pathophysiological mechanisms involved in anthracycline-induced cardiotoxicity [61]. Another factor that plays an important role in the heterogeneity of the reported data is the kinetics of the CTAC and timing of the blood sampling. Advani et al. showed that elevated hs-cTn levels could be measured within 24–48 h after the treatment with doxorubicin in breast cancer patients [36]. Elevated cTn levels were also measured after 3 weeks in all patients (11/11) treated with doxorubicin and in 7/11 patients treated with trastuzumab. Other studies most frequently reported significant cTn increase between 42 and 154 days following the start of anthracyclines [31–35, 38, 39]. Nonetheless, different timing may be expected when it comes to ICI treatments. In most cases, IRCAE and therefore probable cTn elevations occur as early as few hours after the initial dose, up to 60 days after the initial treatment (median 30 days), as reported by Salem et al. [50]. Generally, clinical trials have set follow-up periods with scheduled echocardiography and blood sampling, with additional follow-ups if patients develop signs and symptoms of heart failure. In other words, we are considering cTn values that were quite randomly assayed, likely resulting in a lack of clear appreciation of what levels should be expected in specific stages of CTAC. Finally, a potentially important difference may be between cTnT and cTnI. cTnI is a smaller and potentially better membrane-permeable molecule [62]. It is also abundantly expressed in skeletal myopathies resulting in frequently elevated cTnI levels in patients on chronic hemodialysis [28]. A more recent analysis of hs-cTn assays reported that the release of hs-TnT induced by myocardial damage is smaller compared with hs-TnI [63]. As previously mentioned, conditions such as anemia, electrolyte unbalance, weight changes, and kidney dysfunction that are frequently found in the cancer population, are known to influence cTn levels and should be taken into consideration when interpreting cTn levels.

### Natriuretic Peptides, Potential Predictors for CTAC

Natriuretic peptides (NPs) (e.g., BNP and NT-proBNP) are biomarkers of cardiac volume and pressure overload, mostly known for their use to diagnose acute and chronic heart failure [64]. Low BNP/NT-proBNP levels have excellent negative-predictive value, while elevated levels are indicative of the presence of heart failure. Elevated NP levels can also be found in primary pulmonary diseases and shock, atrial fibrillation, LV hypertrophy, valvular disease, and many more conditions—where they have a strong prognostic value [27, 64–66]. Ideally, BNP/NT-proBNP results should be interpreted in consideration with age, renal function, and body mass index, as those parameters affect NP concentrations [65, 67]. While cutoff values for acute heart failure are useful to fulfill the clinical presentation and to confirm the diagnosis of heart failure, exact values in CTAC are still unknown. Therefore, for chemotherapy-induced heart failure, serial sampling might detect small but significant variations that are useful to diagnose early CTAC.

Lenihan et al. focused their research on the utility of circulating cardiac biomarkers in patients receiving anthracyclines [40]. Their findings supported the validity of BNP/NT-proBNP serial measurements in predicting anthracycline-induced CTAC. Both baseline and post-therapy BNP values, in patients that developed cardiac adverse events, were higher than in those without events. Interestingly, cTnI was not associated with worse outcomes. More recently, Bouwer et al. found a statistically significant correlation between BNP/NT-proBNP levels and trastuzumab-induced cardiotoxicity (TIC) in patients with HER2-positive breast cancer [42]. While all patients demonstrated a NT-proBNP rise during the trastuzumab treatment, the difference was significantly higher in patients that developed TIC. They also reported that for every + 10 pmol/l NT-proBNP rise, LVEF declined by approximately 4%. Similarly, Demissi et al. reported that the doubling of NT-proBNP in patients was associated with a 0.7% decline in LVEF at each subsequent visit [43]. Strikingly, NT-proBNP changes were different between different cancer treatment regimen groups. The changes were most evident in patients receiving anthracycline with trastuzumab, where for each NT-proBNP doubling from baseline an up to 1.3% decline in LVEF was observed. However, in patients receiving only trastuzumab, NT-proBNP levels significantly declined in the first 6 months. Zardavas et al. focused on the role of circulating cardiac biomarkers in monitoring the cardiac safety of patients with early-stage HER2-positive breast cancer receiving trastuzumab [32]. The authors reported higher BNP/NTproBNP levels following anthracycline and before trastuzumab treatment in patients that developed TIC. Adjuvant chemotherapy treatments often come in the combination with radiotherapy. Still, only a few studies focused on the evaluation of possible cardiotoxic effects due to radiotherapy. Palumbo et al. measured BNP values in patients with left-sided breast cancer receiving adjuvant radiotherapy [41] and found that BNP levels were significantly elevated and rising in all patients from first, up to 6 months after the start of radiotherapy. Even 12 months after the start of radiotherapy, BNP levels remained significantly above the baseline value. At the end of the follow-up (median of 74 months), none of the patients developed heart failure and 4 patients developed (MI). While elevated BNP values did not correlate with LVEF decline, it strongly advocates the need for long-term follow-up. Furthermore, D’Errico et al. also suggested that BNP/NT-proBNP could have an important role in detecting myocardial injury and cardiac remodeling after radiation treatment in patients with left-sided breast cancer [68]. In contrast to these studies, others are showing no benefit of BNP in predicting CTAC [35, 36, 69].

These contradictory results could be explained by the heterogeneity of the cancer population and treatments used, different timing of sampling and diverse study protocols. Furthermore, the kinetics of BNP/NT-proBNP release in CTAC, just as is the case with cTn, are still unknown. Ponde et al. suggested that only patients who were treated with anthracyclines showed significant changes in circulating NT-proBNP and cTn levels [69]. This indicates that for other cancer treatments, we still do not have the proper observation, prevention, and therefore no treatment options. Versatile pathophysiological mechanisms of different antineoplastic treatments and genetic predisposition to myocardial stress could result in different effects and timing of myocardial injury. Therefore, different sampling timings but also new, sex-specific cutoff points of circulating biomarkers are needed to diagnose CTAC.

## Other Circulating Biomarkers in Cardio-Oncology

Several other circulating biomarkers are also being assessed for their potential use in diagnosing early myocardial injury before LV dysfunction becomes obviously impaired. As previously mentioned, biomarkers are mostly chosen because of their pathophysiological mechanisms. Researchers are focusing on biomarkers with known pathophysiological mechanisms that could potentially fit and explain processes happening during CTAC (Table 2). In addition to the already discussed circulating biomarkers of myocardial stretch and injury, biomarkers of cardiac fibrosis and oxidative stress like MPO, Gal-3, ST2, and GDF-15 are increasingly studied [34, 43, 70, 71]. Those biomarkers are, however, not cardiac-specific and aggregate research data remain inconsistent. Furthermore, several microRNAs were found overexpressed in cardiac tissue after MI predicting heart failure, while others are found overexpressed in both cardiac and tumor tissues [72].

**Table 2:** List of biomarkers used in cardio-oncology studies. Summary of pathophysiological mechanisms of the most common biomarkers used in cardiology and their practical use in cardio-oncology

**Biomarker**	**Pathophysiological mechanism**	**Antineoplastic treatments**	**Clinical presentation**
cTn (TnT, TnI)	Myocardial injury	ANT, ICI, VEGF, CAR-T, RTx	ACS, myocarditis, acute HF, arrhythmias
NT-proBNP	Myocardial strain	AND + HER-2 inh, TKI, PI, possible CAR-T	Acute and chronic HF
MPO	Oxidative stress	ANT + HER-2 inh	Unknown
Gal-3	Fibrosis	ANT	Acute and chronic HF
ST-2	Endothelial strain and fibrosis	ANT	Acute and chronic HF
GDF-15	Ischemia, oxidative stress, strain	ANT	Acute and chronic HF, ACS
micro-RNA	Cell signaling	ANT	ACS, chronic heart failure
D-dimer	Thrombosis	TKI, VEGF-inh	DVT, PE

*cTn* cardiac troponins, *NT* proBNP natriuretic peptide, *MPO* myeloperoxidase, *Gal-3* galactin 3, *GDF-15* growth differentiation factor-15, *ANT* anthracyclines, *ICI* immune checkpoint inhibitors, *TKI* tyrosine kinase inhibitors, *PI* proteasome inhibitors, *CAR-T* chimeric antigen receptor T cells, *VEGF-inh* vascular endothelial growth factor inhibitor, *ACS* acute coronary syndrome, *HF* heart failure, *KF* kidney function, *DVT* deep venous thrombosis, *PE* pulmonary embolism

Measuring microRNA showed some promising results in detecting early CTAC, but predominantly in animal studies. Finally, in the lack of consistent data, we strongly recommend clinicians to consider an integrated biomarker approach, combining circulating biomarkers with imaging methods. This combined technique showed indeed improved specificity, while sensitivity, as expected, dropped [9, 33, 34].

## Future Directions and Conclusions

There is great relevance and need to screen or survey for CTAC with biomarkers as it may reduce the need for invasive diagnostic tools and interventions, and even costs. These biomarkers should be specific, sensitive, accurate, consistent, and reproducible [73]. Providentially, cardiac-specific circulating biomarkers have demonstrated important signals and may be useful as diagnostic and prognostic markers in the assessment of CTAC.

There are conflicting data regarding the utility of circulating biomarkers before, during, and after anti-neoplastic treatments. The inconsistencies between the published studies in this field could be avoided in the future by designing prospective trials with adequate statistical power, long-term follow-up, and systemic measurement of circulating biomarkers.

Furthermore, strategies that integrate circulating biomarkers and cardiac imaging have an incremental value in identifying cancer patients that are at high risk of CTAC. Also, it has been postulated that biomarker levels are age and gender-specific, which highlights the importance of precision medicine as a potential future solution. The focus should be set not only on selecting the most effective biomarkers for different cancer treatments but also on filling up the knowledge gaps, especially when it comes to the mechanism of myocardial injury. This could be done by pre-selecting standardized timing of the sampling, both baseline and during follow-up, which is greatly influenced by the type of cancer treatment regimen, and by detecting new circulating biomarker thresholds.

**Fig. 3:**
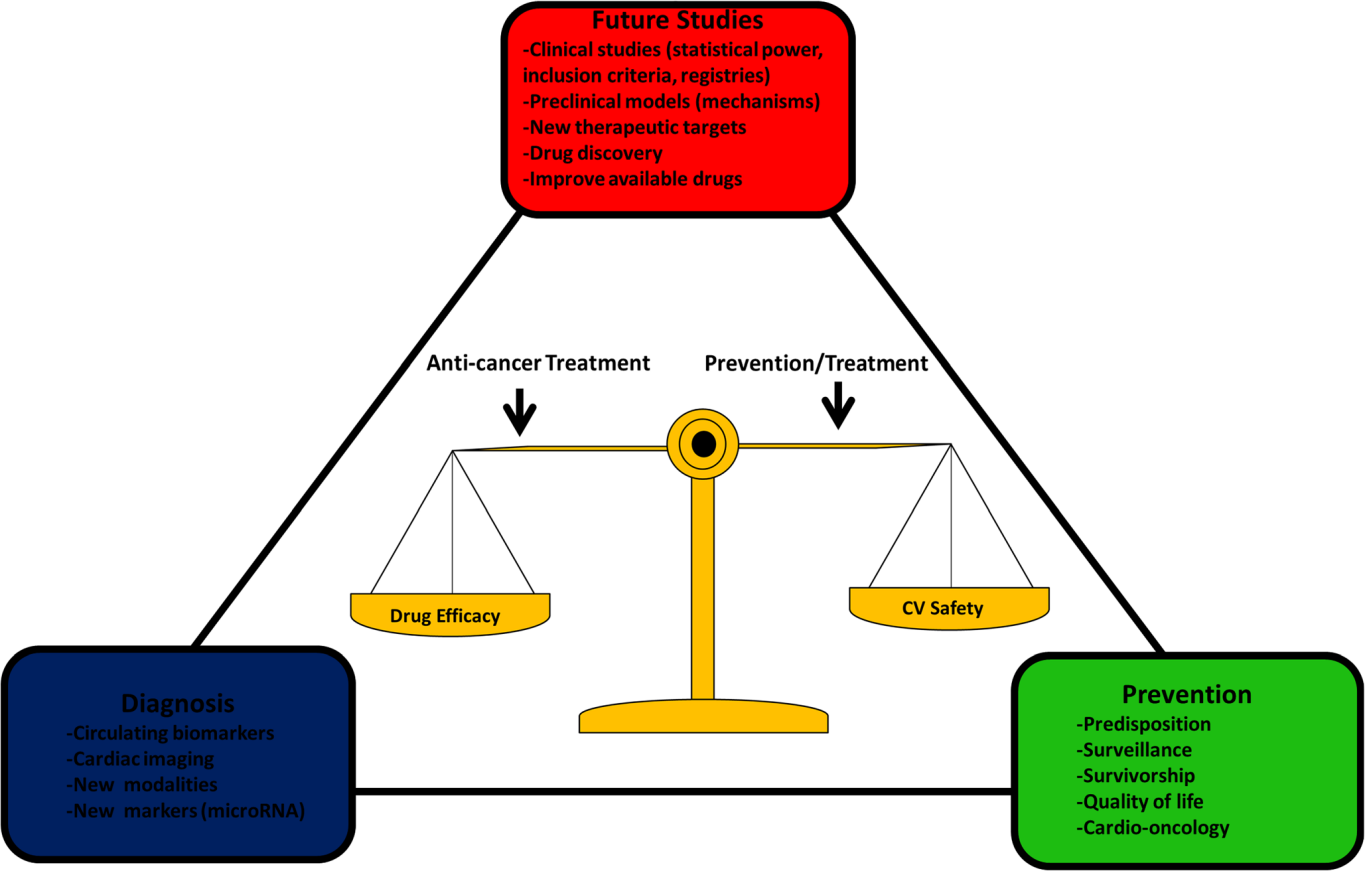
Illustration of future strategies and goals to address the knowledge gap, generate accurate and reliable clinical data, improve diagnostic tools, and optimize treatment and surveillance

Today, most of the available data come from studies that were based on old high-dose treatment regimens and they describe treatments involving only anthracyclines, or regimens that are anthracycline-based. New treatments, such as immune therapy have shown remarkable survival outcomes. However, their long-term effects remain mostly unknown. For a clinician, the current preventive, diagnostic, and treatment options are mostly opinion-based. We would however strongly recommend routine usage of circulating cardiac biomarkers, both cTn and NT-proBNP, and their combination with imaging methods. This way, clinicians can recognize early signs of cardiac damage, and start treatment accordingly. cTn has also shown to have a strong negative predictive value. This may help in recognizing patients that are at low cardiac risk, and steer development of either more aggressive treatment plan, if necessary, or less strict cardiac follow-up. Further, the old definition of cardiotoxicity does not describe all known CTAC effects such as myocarditis, cardiac arrhythmias, or MI following new treatments. Adopting new definitions will most certainly increase the number of patients being diagnosed. The systematization of different cancer treatments and explanations of their pathophysiological mechanisms, leading to updated cardiotoxicity definition are also needed to adapt researchers’ endpoints. This would expand our knowledge, create new clinical approaches, and is likely to improve patient care and survival. Because in the end, a safe continuation of anti-neoplastic treatment is the common goal for everyone—and especially those visiting and working in the cardio-oncology service (Fig. 3).
